# Microwave index engineering for slow-wave coplanar waveguides

**DOI:** 10.1038/s41598-018-24030-w

**Published:** 2018-04-04

**Authors:** Álvaro Rosa, Steven Verstuyft, Antoine Brimont, Dries Van Thourhout, Pablo Sanchis

**Affiliations:** 1 Nanophotonics Technology Center, Universitat Politècnica de València, Camino de Vera s/n, Valencia, 46022 Spain; 2 Photonics Research Group, Department of Information Technology (INTEC), Ghent University–imec, Technologiepark-Zwijnaarde 15, Gent, B-9052 Belgium

## Abstract

Microwave index engineering has been investigated in order to properly design slow-wave coplanar waveguides suitable for a wide range of applications in microwave, photonics, plasmonics and metamaterials. The introduction and optimization of novel capacitive and inductive elements is proposed as a design approach to increase the microwave index while keeping the impedance close to 50 Ω to ensure the compatibility with external electronic devices. The contribution of inductive and capacitive elements and their influence on the performance of the slow-wave coplanar waveguide has been systematically analyzed. As a result, a microwave index as high as 11.6 has been experimentally demonstrated in a frequency range up to 40 GHz which is, to the best of our knowledge, the largest microwave index obtained so far in coplanar waveguides.

## Introduction

Monolithic coplanar waveguides (CPWs) play a key role in integrated devices technology. CPWs can be used for many applications due to their planar geometry (both ground and signal are in the same plane) that reduces the fabrication complexity and makes them compatible with a large variety of structures and applications. Furthermore, CPWs exhibit a very low dispersion, and thus broadband performance, owing to its fundamental quasi-TEM propagation mode^1,2^. Slow-wave CPWs can be viewed as an alternative to regular CPW which allows the slowdown of the propagation velocity as well as the electrical length reduction^3–5^. Therefore, slow-wave CPWs are of paramount importance in several fields such as microwaves, photonics, plasmonics and metamaterials. In the microwave field, slow-wave CPWs are used to design new compact delay lines^6^, phase shifters or microwave filters^7–11^ with an important size reduction in comparison with regular CPWs. In plasmonics, slow-wave CPWs have been used for designing and modelling new spoof surface plasmon modes^12–14^. Moreover the management of the microwave index as well as the impedance is an essential target in metamaterials^15,16^ with several applications like compact multilayer transmission lines, negative and zero order resonator or lens design among others^16,17^. Finally, regarding to the photonic field, slow-wave electro-optic modulators have been reported to reduce the drive voltage and footprint^18–25^. However, slow wave CPWs are required for matching microwave and optical indices to avoid a reduction of the electro-optic modulation bandwidth^22^.

Appropriate tuning of the microwave index of the slow-wave CPW can therefore be beneficial in many fields and applications. The majority of works focus on increasing the capacitance of the CPW as the main method to enlarge the microwave index^9–11,20,23–27^. Here, we propose an improved approach to properly design a high microwave index in slow-wave CPWs by increasing both capacitance and inductance. In such a way, we are able to demonstrate a gradual increase of the microwave index, reaching the highest value reported so far to the best of our knowledge. Furthermore, the influence of the capacitive and inductive elements on the impedance, to ensure a slow-wave CPW compatible with the standard 50 Ω characteristic impedance, is also considered. The proposed slow-wave CPW features broadband performance with a bandwidth extending beyond 40 GHz.

## Microwave theory and proposed design approach

**Figure 1:**
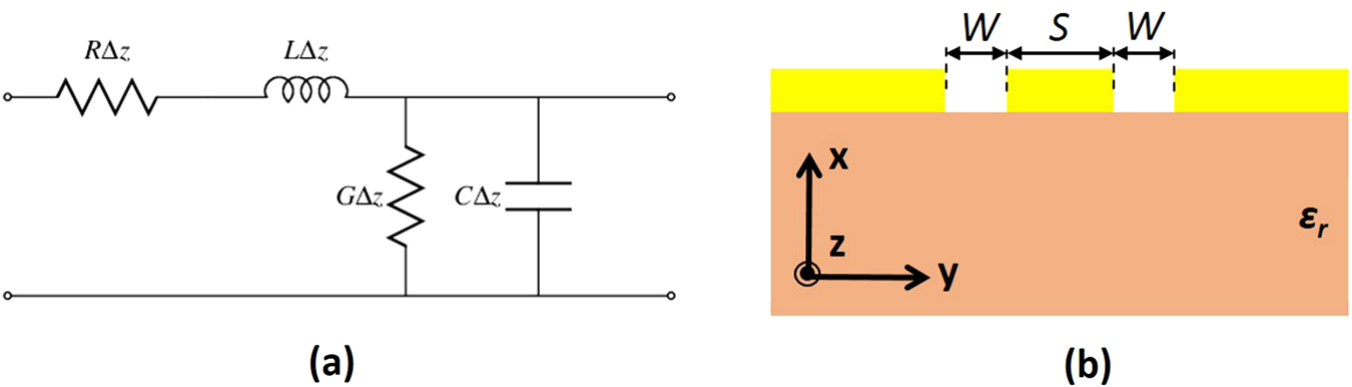
(**a**) Equivalent circuit model and (**b**) transversal view of a CPW.

To address the design of slow-wave CPWs, it is necessary to lay down the basis of microwave theory. This includes basically the transmission line theory to understand the transmission behaviour, and the conformal mapping technique to analyze the influence of the different parameters in a CPW. Figure 1(a) shows the equivalent circuit of a CPW while Fig. 1(b) depicts the transversal view of the CPW with the key design parameters.

**Figure 2:**
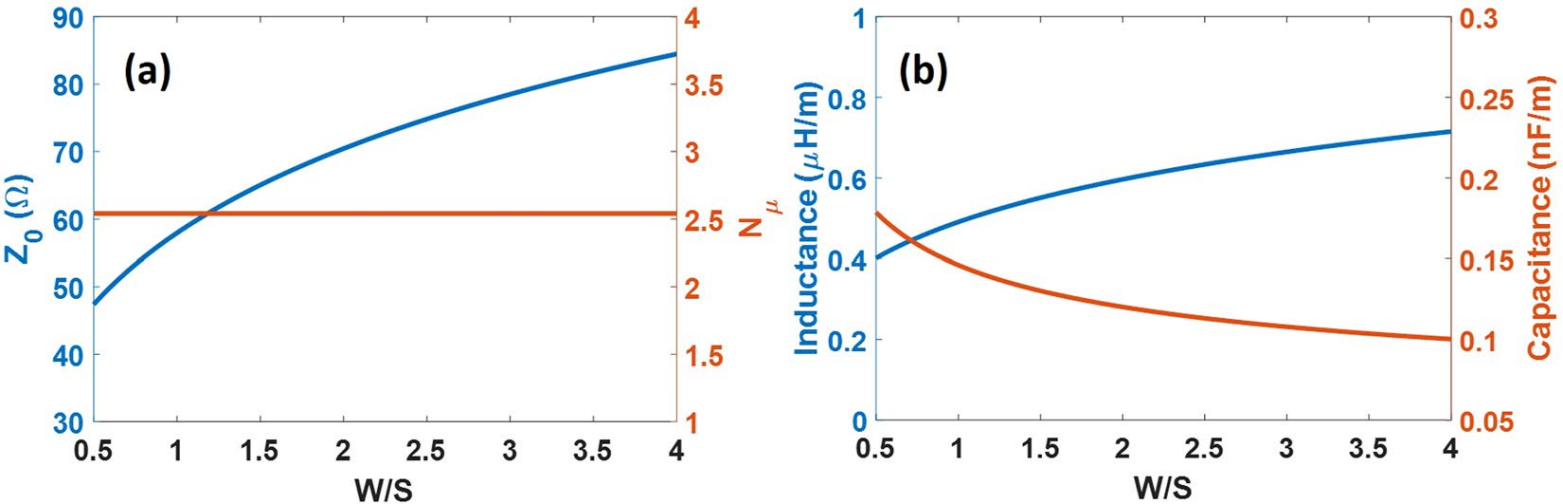
(**a**) Impedance and the microwave index, and (**b**) capacitance and the inductance as a function of W/S obtained by the conformal mapping method.

The CPW has an inductive and capacitive behaviour, as can be seen in Fig. 1(a), so that from the transmission line theory we can obtain the relationship between the microwave index and the impedance with the capacitance, *C*, and the inductance, *L*. That relationship is expressed by the following equations^2^:

1 $${Z}_{0}=\sqrt{\frac{L}{C}}$$

2 $${N}_{\mu }={c}_{0}\sqrt{LC}$$

where *Z*_*o*_ is the impedance, *N*_*µ*_ is the microwave index, *c*_*o*_ is the speed of light in vacuum, *L* is the inductance and *C* is the capacitance. On the other hand, the conformal mapping method links these characteristic parameters of the transmission line with the physical parameters of the CPW^28,29^ like the central strip width or the gap between signal and ground planes, shown in Fig. 1(b):

3 $${C}_{CPW}=2{\varepsilon }_{0}({\varepsilon }_{r}+1)\frac{K({k}_{0})}{K(\sqrt{1-{k}_{0}^{2}})}$$

4 $${L}_{CPW}=\frac{1}{4{c}_{0}^{2}{\varepsilon }_{0}}\frac{K(\sqrt{1-{k}_{0}^{2}})}{K({k}_{0})}$$

where *K(k)* represents a complete elliptic integral of the first kind^29^, *ε*_*r*_ is the relative permittivity of the substrate, *ε*_*o*_ is the vacuum permittivity and

5 $${k}_{0}=\frac{1}{1+2(W/S)}$$

where *W* is the gap and *S* the central strip width of the coplanar waveguide. Therefore, taking into account equations (1)–(5) is possible to obtain the impedance and the microwave index for a regular CPW of given dimensions. Figure 2 shows the influence of *W*/*S* on the impedance and microwave index as well as on the inductance and capacitance for a silicon substrate (*ε*_*r*_ = 11.9).

As can be seen in Fig. 2(a), the impedance increases when *W/S* increases. However, the microwave index remains constant over the whole *W/S* range because the reduction of the capacitance is balanced by the larger inductance (Fig. 2(b)). In previous works^10,11,20,23–26^, the introduction of periodic capacitive elements (thin fins) to the CPW has been proposed to increase the capacitance without decreasing the inductance, which results in a microwave index increase and an impedance reduction. This is an effective method but does not take into account the inductance as a parameter that may also be exploited for the design. In order to manipulate the inductance, the CPW strips can be reduced by using periodic thin slots. The proposed slots allow the inductance to be increased while only having a small effect on the capacitance. Therefore, increasing the inductance can be combined with approaches based on increasing the capacitance. Such a combination will allow us to reaching much higher microwave indices, while keeping the impedance close to 50 Ω.

**Figure 3:**
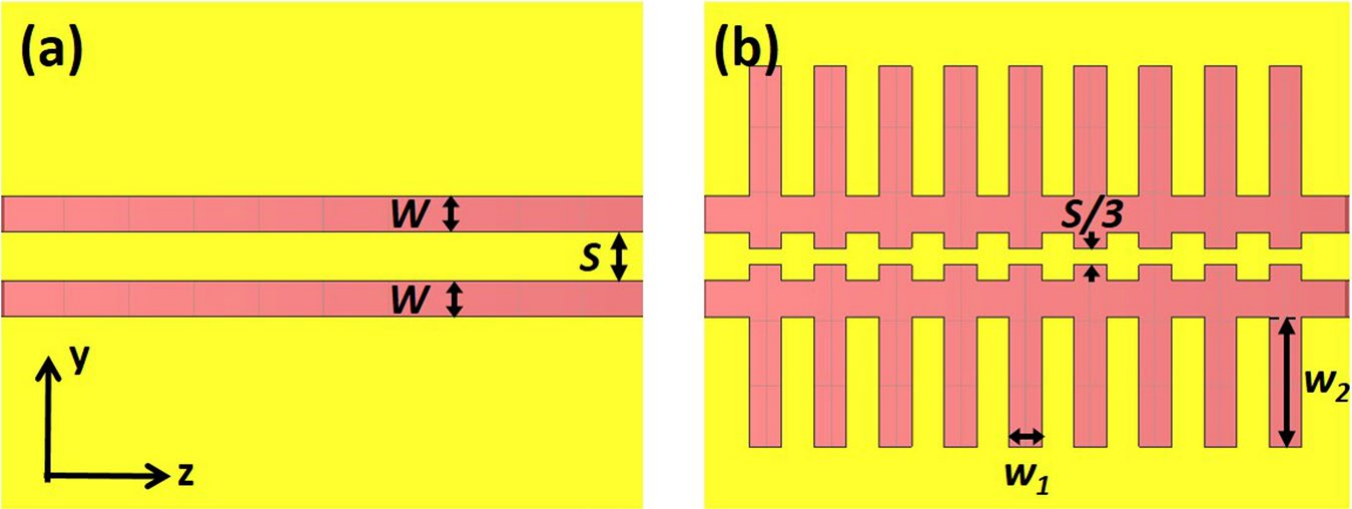
(**a**) Top view of a regular CPW and (**b**) of a slow-wave CPW.

To design the slow-wave CPW, it is crucial to analyze the thin slots in order to understand their influence on the inductance and the capacitance and therefore on the microwave index. The analysis has been carried out using the electromagnetic simulation software CST microwave studio. CST is a simulation tool that solves Maxwell equations for each point on a 2D or 3D mesh using a finite elements method. The frequency has been fixed at 20 GHz. Figure 3 shows a top view of the regular and slow-wave CPWs with the parameters to be designed. For the regular CPW, *S* = 15 µm and *W* = 11 µm so that *W/S* = 0.73. By properly checking Fig. 2(a), an impedance value close to 50 Ω is achieved.

**Figure 4:**
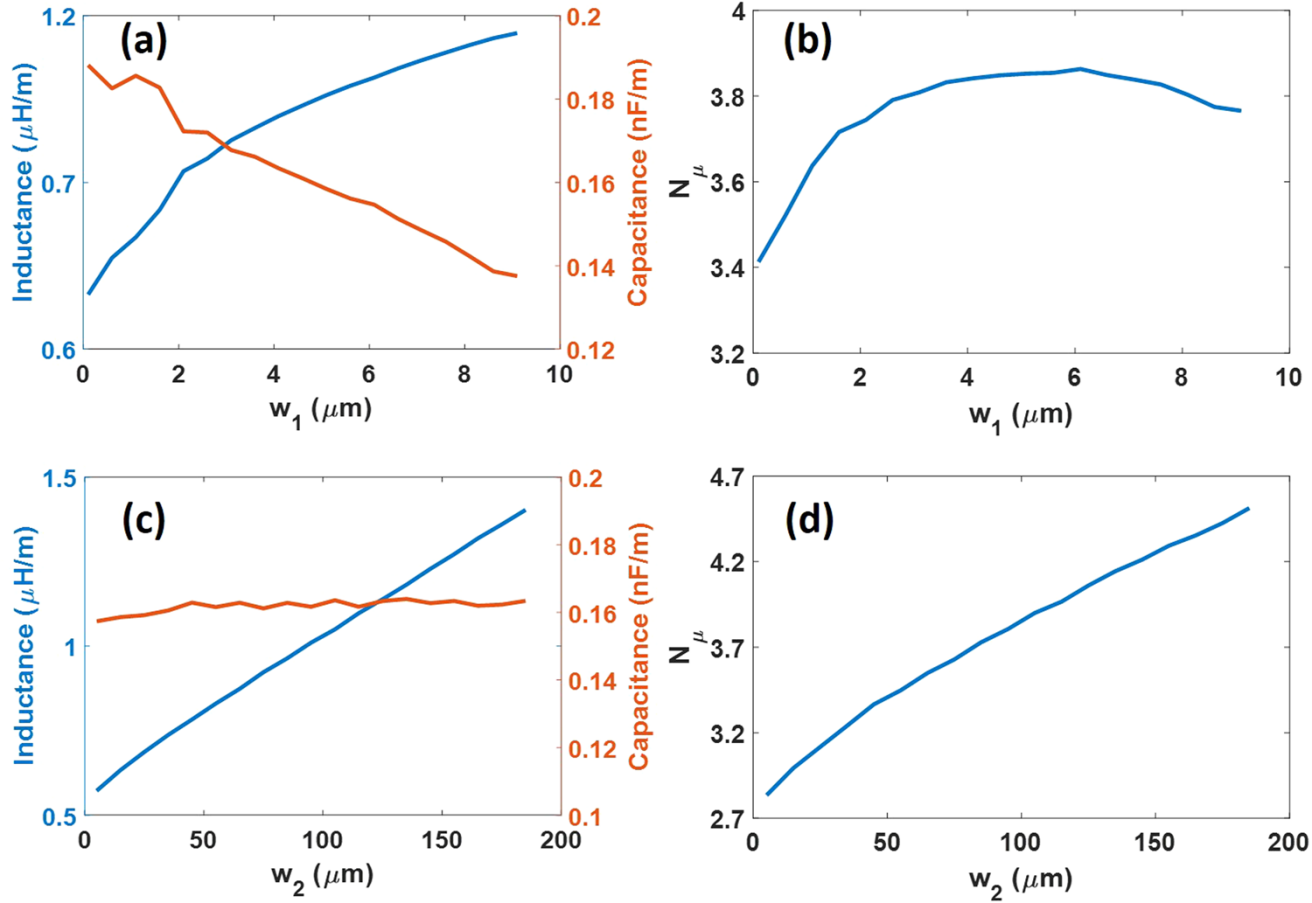
(**a**) Simulated inductance and capacitance and (**b**) microwave index as a function of w_1_ and w_2_ = 100 µm and (**c**) simulated inductance and capacitance and (**d**) microwave index as a function of w_2_ and w_1_ = 5 µm for the slow-wave CPW shown in Fig. 3(b).

Figure 3(b) depicts the proposed slots to increase the inductance, where *w*_1_ and *w*_2_ are the length and width of the slot. The period of the slots has been previously optimized to 20 µm. The variation of the capacitance and inductance as a function of *w*_1_ is shown in Fig. 4(a) for *w*_2_ = 100 µm. It can be seen that there is an opposite behavior between them. The increase of the inductance is higher than the capacitance reduction, resulting in a microwave index increment (Fig. 4(b)). However, the increase of the inductance is not constant with *w*_1_ so that the increase of the microwave index is reduced for larger *w*_1_ values. On the other hand, the behavior of the capacitance and the inductance with *w*_2_ is shown in Fig. 4(c), for *w*_1_ = 5 µm. While the inductance increases with *w*_2_, the capacitance remains constant, which gives rise to higher microwave index with larger *w*_2_, as it can be seen in Fig. 4(d).

**Figure 5:**
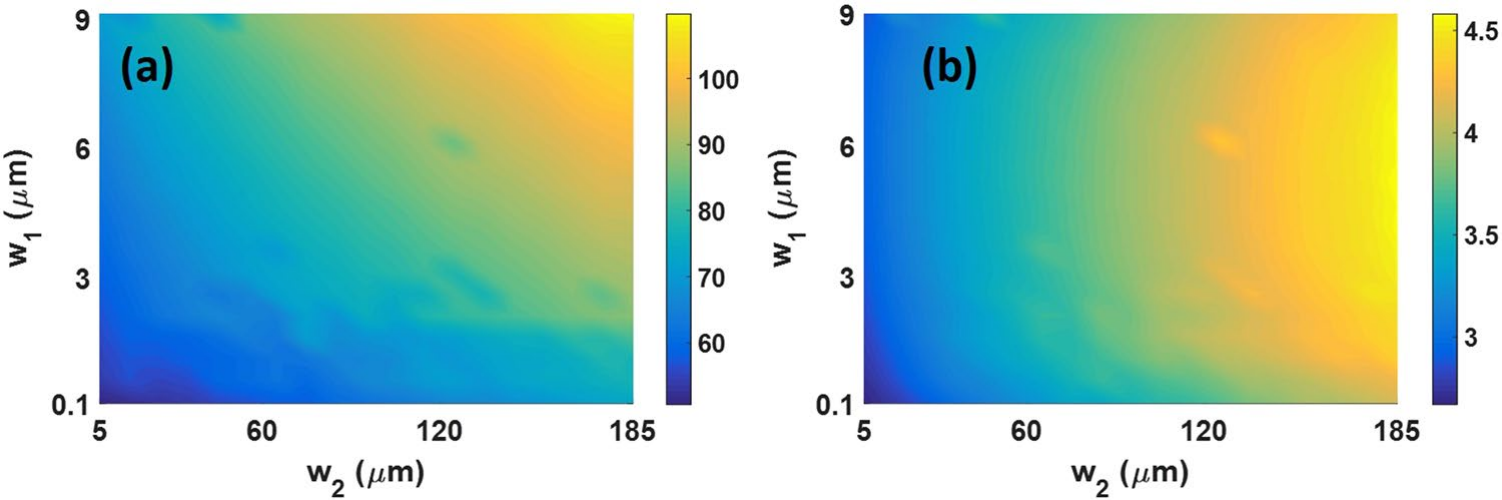
(**a**) Impedance and (**b**) microwave index as a function of w_1_ and w_2_.

**Figure 6:**
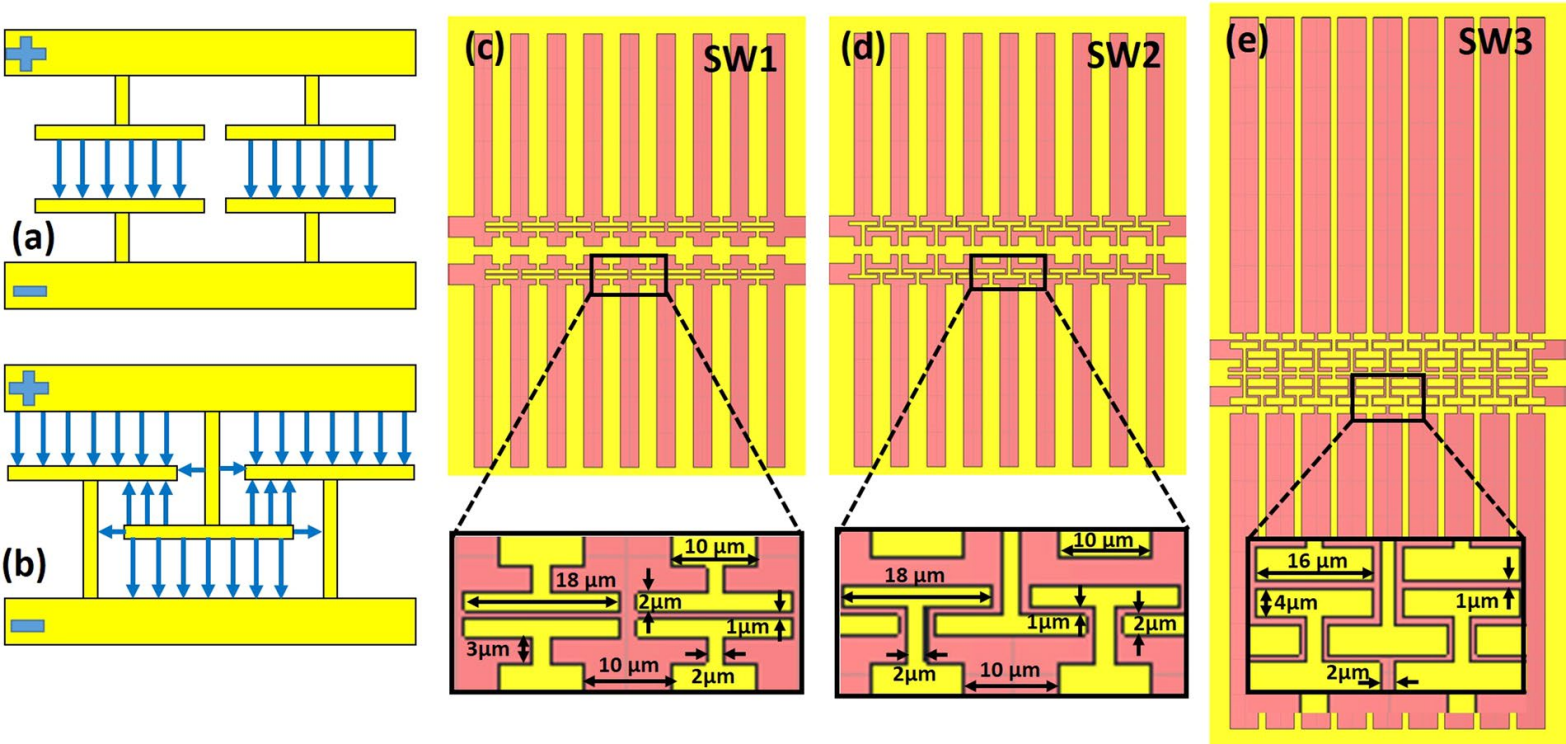
Schematic of (**a**) parallel and (**b**) novel crossed T-rails to increase the capacitance of the CPW. The blue lines represent the induced electric field due to the T-rails. Slow-wave CPW with (**c**) parallel T-rails, (**d**) crossed T-rails and (**e**) a combination of both configurations for improved performance.

Figure 5(a) shows the impedance as a function of *w*_1_ and *w*_2_. The corresponding microwave index is depicted in Fig. 5(b). It can be seen that larger microwave indices are achieved at the expense of increasing the impedance, which is in agreement with equation (1) as the inductance increase dominates over the capacitance. Therefore, it is clear that the proposed slots act as enhanced inductive elements in the slow-wave CPW. However, to keep the impedance close to 50 Ω without reducing the microwave index, the introduction of capacitive elements is also required. The capacitance can be increased with the introduction of parallel T-rails, as shown in Fig. 6(a), which are periodically repeated along the propagation direction^21–24^. A novel approach based on a crossed T-rail, as depicted in Fig. 6(b), is here also proposed to further increase the capacitance. The induced electric field due to the T-rails is represented with blue lines in Fig. 6(a,b).

Parallel T-rails with a length of 18 µm, width of 2 µm and gap of 1 µm have been added on the inductive slow-wave CPW with *w*_1_ = 5 µm and *w*_2_ = 100 µm. The slow-wave CPW is shown in Fig. 6(c) and has been named as SW1. The same inductive slow-wave CPW but with the crossed T-rails has also been considered to evaluate the difference in capacitance and so the influence on the microwave index. In this case, the CPW is depicted in Fig. 6(d) and has been named as SW2. Finally, a slow-wave CPW with a combination of both crossed and parallel T-rails, named as SW3 and shown in Fig. 6(e), has also been designed to further improve the capacitance. The length and width of the T-rails have been optimized to 16 µm and 4 µm, respectively. Furthermore, the inductive performance has been enhanced by changing *w*_1_ to 17 µm and *w*_2_ to 180 µm. As the capacitance of the slow-wave CPW is ruled by the capacitive T-rail elements, the reduction of the capacitance due to the larger *w*_1_ is negligible.

**Figure 7:**
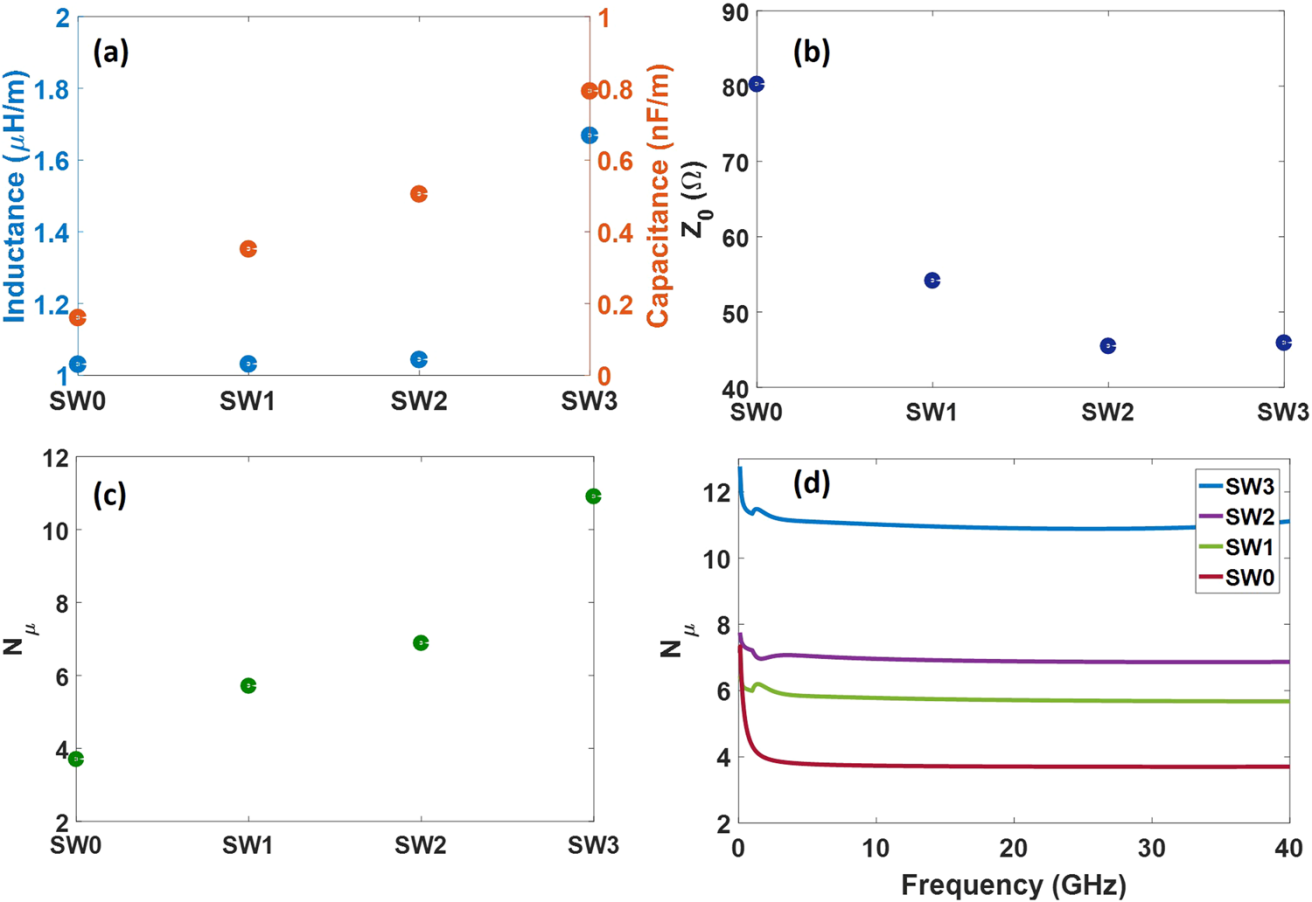
(**a**) Inductance and capacitance, (**b**) impedance and (**c**) microwave index for the different designs of the slow-wave CPWs obtained by simulations at 20 GHz. (**d**) Simulated microwave index as a function of the frequency.

Figure 7 shows the simulation results for the different designs of the slow-wave CPWs. SW0 refers to the inductive slow-wave CPW shown in Fig. 3(b) with *w*_1_ = 5 µm and *w*_2_ = 100 µm. The capacitive and inductive behavior of the slow-wave CPWs are depicted in Fig. 7(a). The capacitance is gradually improved up to a factor of four (from 0.2 to 0.8 nF/m) by the introduction of the capacitive T-rail elements in SW1 and their modifications in SW2 and SW3. The inductance is approximately constant for SW0, SW1 and SW2, but it increases from 1.05 to 1.67 µH/m for SW3 due to the changes in the ground slots and the modification of the signal strip. The impedance, Fig. 7(b), and microwave index, Fig. 7(c), will be determined by the capacitive and inductive behavior. The impedance is decreased to a value close to 50 Ω for the improved designs of the slow-wave CPWs due to the larger capacitance with respect to the original design of SW0. The microwave index is also increased for SW1 and SW2 due to the larger capacitance but the improvement is much larger for SW3 due to the additional increase of the inductance. Thereby, the microwave index is significantly enhanced from 6.9 in SW0 up to 11 in SW3. The frequency response has also been simulated and is shown in Fig. 7(d). A constant microwave index and therefore wideband operation is achieved due to the low dispersion of the quasi-TEM propagation mode in the slow-wave CPW.

## Characterization and Conclusions

**Figure 8:**
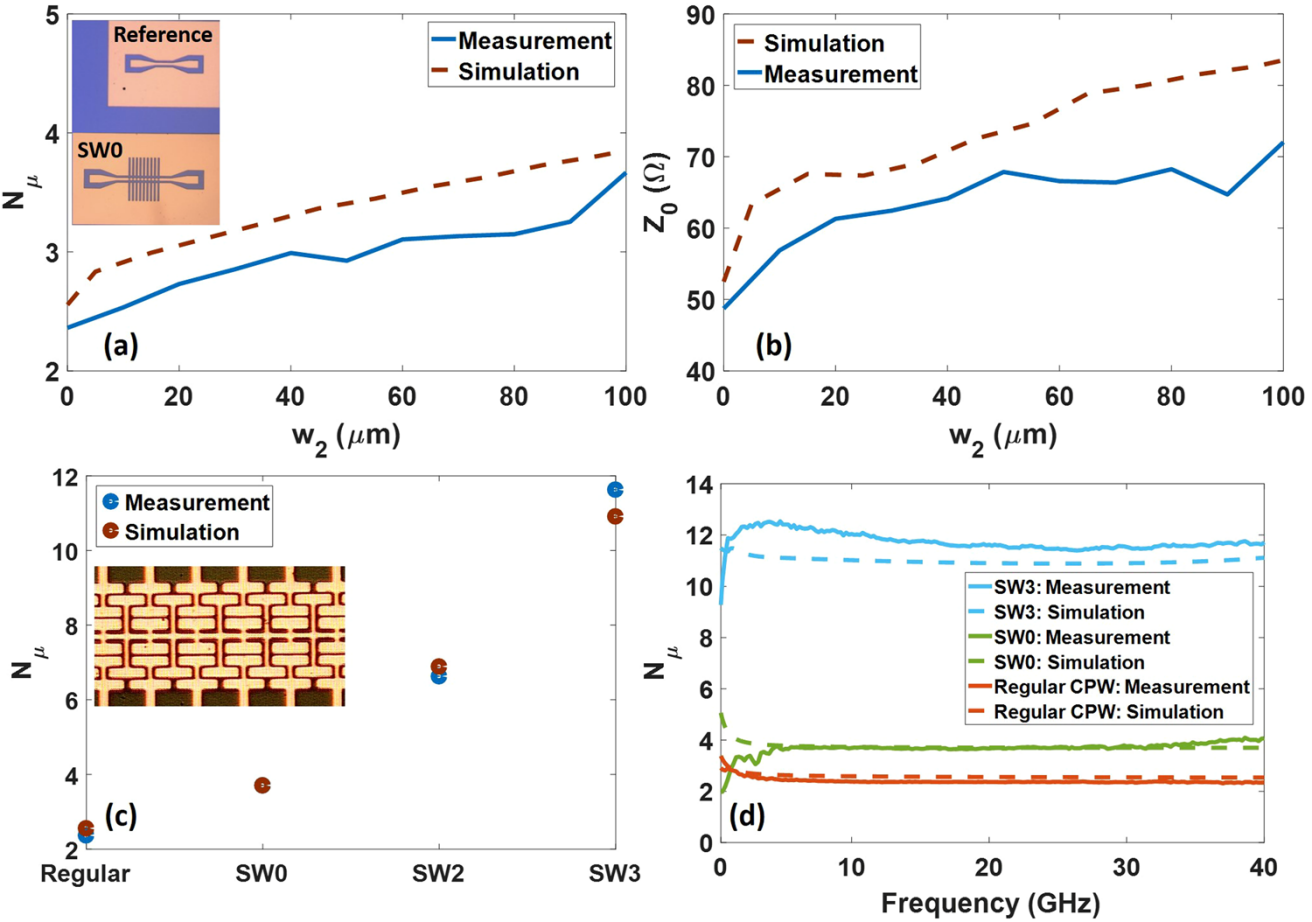
(**a**) Simulated and measured microwave index and (**b**) impedance for SW0 as a function of w_2_ and assuming w_1_ = 5 µm. An inset with an optical view of the reference CPW and SW0 with w_2_=100 µm is included. Simulated and measured microwave index (**c**) at 20 GHz for the regular CPW, SW0, SW2 and SW3 and (**d**) as a function of frequency for the regular CPW, SW0 and SW3. The inset in (c) shows an optical view of the fabricated SW3.

In order to demonstrate the simulated performance, some of the previously designed slow-wave CPWs have been fabricated and characterized. The devices were fabricated on a silicon substrate covered with a 300 nm thick SiO_2_ layer deposited using a plasma enhanced chemical vapor deposition (PECVD) process. The CPWs where formed with a lift-off process using TI35E photoresist in an image reversal process. The electrodes consists of 40 nm Ti, deposited through sputtering and 1000 nm Au, deposited through thermal evaporation. Figure 8 shows the obtained measurements results and the comparison with simulations. A multiline method has been applied to extract the microwave index from the slow-wave part of the CPW. The method uses a reference CPW (inset of Fig. 8(a)) in addition to the slow-wave CPW. A more detailed description can be found elsewhere^30,31^.

Figure 8(a,b) show the variation of the microwave index and impedance in SW0 for values of *w*_2_ varying from 10 µm to 100 µm, and the regular CPW (Fig. 3(a)), represented at w_2_=0 µm. It can be seen that there is a good agreement with simulations. Figure 8(c) shows the measured microwave index for the regular CPW, SW0, SW2 and SW3. Also these results are in very good agreement with the simulations. The microwave index is improved from 2.36, 3.7 and 6.9 for the regular CPW, SW0 and SW2, respectively, up to 11.6 for SW3. An inset of Fig. 8(c) shows an optical image of the fabricated SW3. Additionally, Fig. 8(d) depicts the frequency response measured for a range up to 40 GHz.

Table 1 compares the obtained results in terms of microwave index, propagation losses and impedance with the values reported in the last two decades for slow-wave CPWs. The microwave index is largely increased for SW3 with respect to previous works. Furthermore, though propagation losses increase for SW3 with respect to SW0 and the regular CPW, the value remains comparable and even lower than the ones achieved in CPWs with an impedance of around 50 Ω.

**Table 1:** Microwave index, propagation losses and impedance comparison for reported slow-wave transmission lines.

Reference	Year	Structure	Nµ	α(dB/mm)	Z_0_ (Ω)
^5^	2005	CPW	4.47	4–6.9	50
^9^	2013	CPW	5.3	0.7	35
^11^	2001	CPW	3.74	—	50
^19^	1994	CPW	3.15	40	50
^20^	2017	CPW	3.9	2	50
^21^	1996	CPW	2.6	46	50
^23^	2011	CPW	4	33	50
^24^	1993	CPW	3.4	—	50
^25^	1995	CPW	3.37	2	45
^26^	2009	CPW	5.9	0.4	43
^27^	2015	CPW	7.38	1.4	34
This work (CPW)	2018	CPW	2.5	0.25	50
This work (SW0)	2018	CPW	3.7	0.6	80
This work (SW3)	2018	CPW	11.6	5.4	50

In summary, we have demonstrated a design approach to increase the microwave index in CPWs. The proposed approach is based on the design of periodically distributed inductive and capacitive elements. A microwave index of 4.7 has been achieved by increasing the inductive behavior of the CPW with small slots on the ground and signal planes. In addition, the effect on the impedance has also been considered and it has been shown that it is possible to achieve high microwave indices while keeping the impedance around 50 Ω. Therefore, through the combination of inductive and capacitive elements, a microwave index up to 11.6 has been demonstrated. To the best of our knowledge, such value is the largest to date obtained in planar transmission lines.

### Data availability

Requests for materials should be addressed to A.R.
